# Effects of a Novel Formulation on Oral Biofilm, pH Buffering, and Gingival Health in Patients with Dry Mouth

**DOI:** 10.1155/2018/2748274

**Published:** 2018-09-23

**Authors:** Thair Takesh, Jessica Ho, Miracle Vania Firmalino, Delaney Islip, Afarin Anbarani, Petra Wilder-Smith

**Affiliations:** Beckman Laser Institute, University of California, Irvine, CA 92612, USA

## Abstract

**Goal:**

To identify in patients with dry mouth the effects of a novel test agent (Oral Essentials Hydrating Formula Mouthwash, Beverly Hills, CA) versus a control agent (Biotène Dry Mouth Oral Rinse, GlaxoSmithKline Consumer Healthcare L.P., Moon Township, PA, USA) versus no treatment on dry mouth, plaque, salivary pH and buffering capacity, gingival health, and tooth sensitivity.

**Materials and Methods:**

In this cross-over study, ten subjects with dry mouth used test and control dry mouth interventions, as well as no dry mouth intervention in randomized sequence. Plaque Index, Gingival Index, Sulcus Bleeding Index, Plaque staining, and photographs were recorded at baseline and end of each study arm. Salivary volume, pH, and buffering capacity were also recorded at these time points. Additionally, subjects completed a questionnaire for dry mouth and dentinal sensitivity at each visit.

**Results:**

Reductions in plaque presence and clinical indices were similar after use of test or control products (*p* < 0.05). Saliva volume and pH buffering improved significantly after use of test and control products (*p* < 0.05).

**Conclusions:**

The effects of a novel dry mouth intervention are similar to those of an existing OTC remedy and are significantly better than no intervention.

## 1. Introduction

With a reported prevalence of 5% to 46% [1], dry mouth has many negative sequellae [2, 3]. As saliva lubricates and cleanses the mouth, protects teeth through its buffering and remineralizing properties, supports antimicrobial activity and hard tissue remineralization [4], and assists with chewing and speech, adequate salivation is essential to oral health and comfort. Inadequate saliva presence is more common in the elderly. One study reported persistent dry mouth in 17.5% of the elderly surveyed, with significantly higher prevalence in women [5]. With increasing age, chronic conditions that can cause dry mouth become more common, and the associated medications can further exacerbate the overall reduction in salivary presence, as well as variations in its biochemical composition and functions [6–15]. Other causes of dry mouth include head and neck radiotherapy [16], salivary gland disorders [17], diabetes [17], and Sjögren's syndrome [18].

Dry mouth symptoms range from mild discomfort to considerable oral disease that may impact oral health and even the patient's quality of life [3]. Oral and dental consequences include sensations of soreness and burning, difficulties in speaking, chewing and swallowing [17], mucosal atrophy [19], poor denture retention [20], and an altered oral microbiome [20–25]. Moreover, a significantly higher prevalence of enamel demineralization, dentinal sensitivity, and progressive dental caries, as well as periodontal disease is reported in patients with chronic dry mouth [20].

Treatment for dry mouth typically takes 2 forms: (1) addressing etiological factors and (2) alleviating symptoms and/or increasing salivary flow [1, 17]. Systemic medications to stimulate saliva secretion include pilocarpine and cevimeline [26]. Moreover, a wide range of over-the-counter agents are available, including mouthwashes, rinses, sprays and gums, or salivary substitutes. Nevertheless, dry mouth remains an inadequately managed, common and chronic complaint, especially among the geriatric population [27].

The goal of this clinical study was to identify, in patients with dry mouth, the effects of (1) a novel test agent (Oral Essentials Hydrating Formula Mouthwash, Beverly Hills, CA) versus (2) a control agent that is commonly used by individuals with dry mouth (Biotène Dry Mouth Oral Rinse, GlaxoSmithKline Consumer Healthcare L.P., Moon Township, PA, USA), and (3) no treatment on plaque presence, salivary pH and buffering capacity, gingival health and tooth sensitivity, as well as intraoral comfort and function. These goals were formulated to test the hypothesis that the novel mouthwash has similar effects on these variables as one of the leading dry mouth agents, Biotène Dry Mouth Oral Rinse.

## 2. Materials and Methods

### 2.1. Human Subjects

This study was performed in full compliance with University of California IRB-approved protocol 2013–9778. All work was conducted in accordance with the Declaration of Helsinki (1964), with the human subjects' understanding and written consent. Ten subjects with confirmed dry mouth participated in this study. They were recruited at the University of California, Irvine by word-of-mouth, flyers, and e-mail recruitment. All subjects signed an informed consent at the beginning of the study, as well as statement of patient rights and photographic release forms. Subjects received an incentive payment at study completion.

#### 2.1.1. Subject Inclusion/Exclusion Criteria

  Inclusion criteria:
  (1) Males and female subjects of all races and ethnicities, age between 18 and 75 years old
  (2) Demonstrated an unstimulated whole saliva flow rate below 0.2 ml per minute and a stimulated saliva flow rate less than 0.5 ml in 5 minutes
  (3) At least 5 natural teeth present in each quadrant (excluding third molars)
  Exclusion criteria:
  (1) Participation in any other clinical study involving the mouth or xerostomia within the last 30 days prior to enrolment into this study.
  (2) Pregnant or nursing women (self-reported).
  (3) Subjects who were unable to defer dental treatment during the study dates.
  (4) History of significant adverse effects following use of oral hygiene products such as toothpastes and mouth rinses or allergy to personal care/consumer products or their ingredients.
  (5) Significant past unresolved or current medical problem history.
  (6) Presence of other major pathologies, such as herpetic infection, major recurrent apthous ulcers, or other ulcer forming diseases, abscesses, granulomas, or severe gingivitis, which might compromise the ability to perform measurements.
  (7) Other significant disease or disorders that, in the investigator's opinion, would exclude the subject from the study including systemic conditions that would influence the course of periodontal disease.
  (8) Active acquired immunodeficiency syndrome (AIDS) or hepatitis B/C (self-reported).
  (9) Smokers.
  (10) Self-reported GERD condition.
  (11) Presence of any condition, abnormality or situation at baseline that in the opinion of the Principal Investigator night preclude the volunteer's ability to comply with study requirements, including completion of the study or the quality of the data.
  Subject restrictions:
  (12) Subjects would not be allowed to receive dental treatment (except emergency treatment) during the study.
  (13) Subjects would be asked to refrain from all nonstudy oral hygiene procedures other than their usual brushing, flossing, and mouth washing routine during the study.
  (14) Subjects embarking on a course of medication during the study would have to inform the Study Coordinator so that a decision could be made as to whether they could continue in the study. A five-minute unstimulated saliva test might be taken to verify no change in saliva flow resulting from use of the new medication. Compliance with the protocol was checked at each test visit and recorded on the appropriate documentation.

### 2.2. Protocol

**Figure 1:**
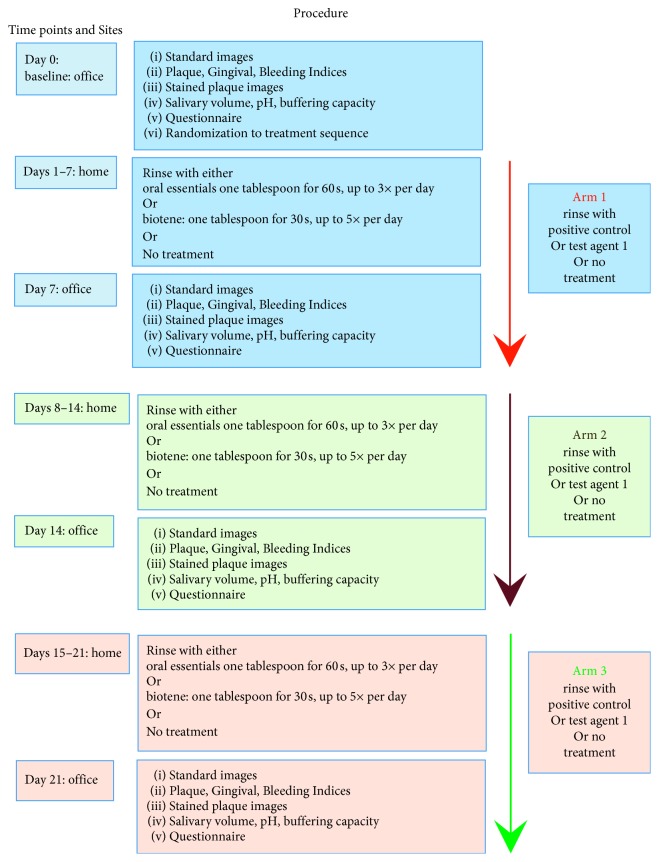
Flow chart of study protocol.

The study had 3 arms, whereby in two arms, subjects used a test or control dry mouth intervention; in the third arm, they used no intervention for xerostomia (Figure 1). Subjects were prerandomized with regard to interventional sequence using online randomizer software (Research Randomizer software: https://www.randomizer.org/). Test and control formulations were provided in numbered plain white containers to blind subjects and investigators with regard to treatment allocation. For the “no treatment” category, subjects were asked to refrain from any symptomatic dry mouth treatment; therefore, in this section of the study, the patient was not blinded. However, the clinical evaluator remained blinded as to the treatment allocation. Subjects were provided with a new standard Oral B ProFlex toothbrush (Procter & Gamble, Cincinnati, OH, U.S.) for each arm of the study and instructed to continue with their usual oral hygiene measures.

After obtaining informed written consent during the baseline (Day 0) visit, standardized photographs as well as full-mouth Plaque Index (PI) [28], Gingival Index (GI) [29], and Sulcus Bleeding Index (mSBI) [30] were recorded for all teeth by an experienced clinician precalibrated to 95% consistency for all 3 indices in 100 periodontal patients over the past 6 months. Plaque was stained (2-Tone Disclosing Agent, Young Dental, Earth City, MO) and photographed at baseline and at the end of each study arm. Standardized photographs of the buccal/labial surfaces of all teeth were recorded by the same clinician using a Nikon D3200 camera with 18–55 mm lens and ring flash. A dental photographic mirror was used as necessary to visually access all surfaces. Salivary volume, pH, and buffering capacity were also determined. Saliva was collected during office visits at baseline and at the end of each study arm by asking the seated subject to pool saliva in the floor of their mouth for 5 minutes, then to expectorate the saliva into a sterile graduated collecting cup. Subjects recorded daily how many times they used each product. Additionally, they completed a standardized self-evaluation questionnaire for dry mouth, dentinal sensitivity, and product evaluation at each visit (Figure 1).

### 2.3. Products and Method of Use

#### 2.3.1. Test Product

Oral Essentials Hydrating Formula Mouthwash, Beverly Hills, CA, USA. Use approximately one tablespoon, swish vigorously for 60 seconds, and then spit out. Use up to 3 times per day.

#### 2.3.2. Control Product

Biotène Dry Mouth Oral Rinse was obtained from GlaxoSmithKline Consumer Healthcare L.P., Moon Township, PA, USA. Use approximately one tablespoon, rinse for 30 seconds, and then spit out. Use up to 5 times per day.

### 2.4. Data Analysis

Image J software (https://imagej.nih.gov/ij/) was used to process the digital intraoral photographs of the buccal/labial surface of each tooth, subject, and timepoint. This was achieved using the standard technique, whereby the borders of all plaque accumulations are visually delineated, and the areas thus mapped are expressed as percent coverage of each tooth surface. Digital image analysis also evaluated plaque age at each location, based on stain color (red stain indicates new plaque less than 24 h in age; blue stain indicates old plaque more than 24 h in age).

The value for each clinical index for each tooth unit at the end of each washout period was used as the baseline value for the subsequent arm of the study. The effects of each dentifrice on each clinical index and on plaque presence were tested using sums and differences of changes between study arms, calculated for each subject. Means and standard deviations were calculated for each group. The sums and differences were tested for significance by means of a two-sample *t*-statistic. A two-sample *t*-test was also performed on the differences in the changes in each arm to see whether one treatment was more effective than the other.

## 3. Results and Discussion

### 3.1. Subject Questionnaire

**Figure 2:**
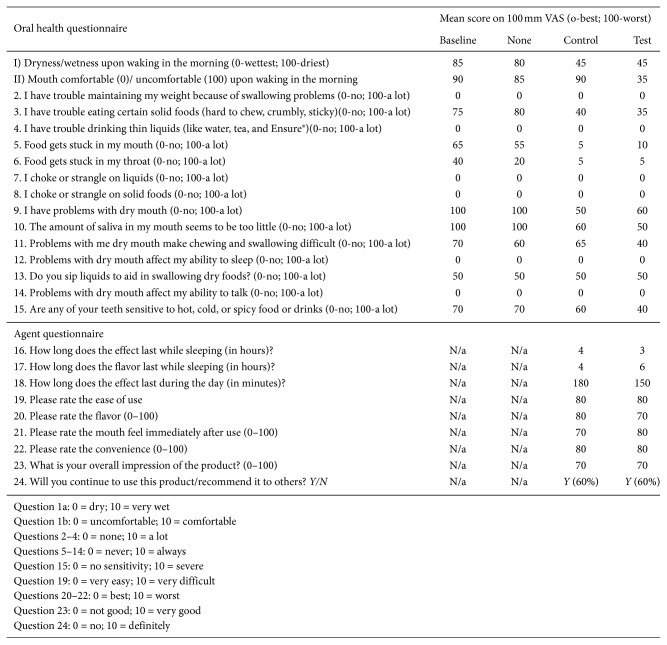
Questionnaire self-evaluation responses.

All subjects completed a routine self-evaluation questionnaire for dentinal sensitivity, dry mouth status, and mouthwash ease of use at every visit (Figure 2).

#### 3.1.1. Oral Health Questionnaire

Clinical criteria showed outstanding and highly significant (*p* < 0.01) improvement during the test and control agent use arms of the study except for “oral comfort at waking in the morning” (significant effect only for test intervention) and “do you sip liquids to aid swallowing” (no significant change from baseline for test and control interventions).

#### 3.1.2. Agent Questionnaire

Overall, subject response was very positive. Ease of use, flavor, and mouth feel all scored highly at >70/100. Almost 80% of subjects stated that they would prefer a longer-lasting product for test and control products.

Overall, functions related to eating, sleeping, and overall oral comfort were improved using either intervention versus no intervention. This seems reasonable, as both the test and control formulations target symptomatic relief rather than long-term physiological functions. The level of symptomatic improvement is similar to that reported in other studies [28].

### 3.2. Plaque

**Figure 3:**
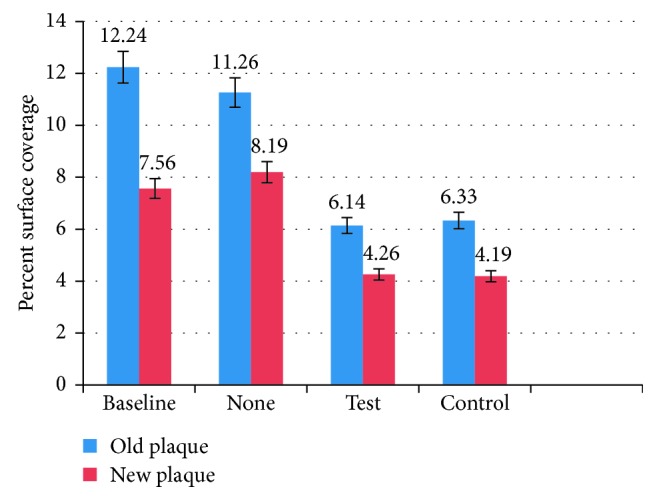
Mean (SD) plaque presence at baseline and after each 7-day arm of the study, expressed as % of image surface area covered by old (>24 h) and new (<24 h) plaque, respectively.

Surface plaque was quantified three times: at baseline, and after each 7-day study arm, respectively (Figure 3). Plaque on oral surfaces was stained using a plaque-disclosing solution (2-Tone Disclosing Agent, Young Dental, Earth City, MO), and standardized intraoral photographs were recorded to document the extent of the plaque staining on all natural teeth. Using image J software, two assessments were made:

- Plaque age based on stain color (blue vs red)
- Percent of total tooth surface covered by plaque

Using Image J software, % of surface coverage was quantified for old (>24 h) and new (<24 h) plaque for each study arm.

There was a significant reduction in old and new plaque presence after use of test or control products for 1 week versus no product (*p* < 0.05). Old and new plaque levels were similar for the test and the control products (*p* > 0.1). Old plaque presence fell to approximately 50% of the baseline value, while new plaque presence was reduced to approximately 60% of the baseline level. Typically, greater saliva presence and flow are associated with better lubrication and self-cleansing of the mouth [31], which are both linked to reduced plaque presence as well as easier and more effective oral hygiene, so this result is not unexpected.

### 3.3. Clinical Indices

**Figure 4:**
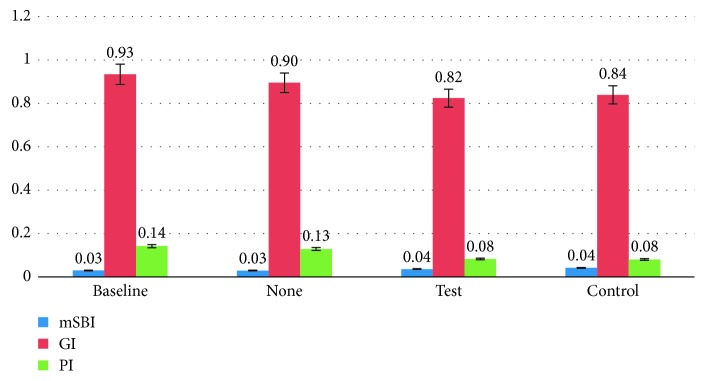
Mean clinical indices (SD) at baseline, and after each 7-day arm of the study.

Clinical indices were quantified three times: at baseline, and after each 7-day study arm, respectively, using 3 standard numerical scales: Plaque Index (Quigley-Hein, Turesky Modification Plaque Index) (PI), Gingival Inflammation (Loe and Silness Gingival Index) (GI), and Gingival Bleeding (mSBI) (Figure 4).

Generally, clinical indices were similar for the test and the control products (*p* > 0.1). There was a significant reduction in clinical Plaque Index by approx. 60% after use of test or control products for 1 week (*p* < 0.05). This parallels the reduction in clinical plaque presence that was quantified using photographs and image quantification software (see previous paragraph). The reduction in Gingival Index after 7 days of test or control product use almost reached a significant level (*p*=0.0563 for test product, *p*=0.0643 for control product). A lower Gingival Index is generally seen as an indication of better gingival health. This finding may be related to the reduction in plaque levels, which are typically related to an improvement in gingival health [32]. Because the duration of each study leg was relatively short, it is not possible to determine whether longer-term usage of the control or test interventions would have resulted in further improvements in the Gingival Index. The Sulcus Bleeding Index (mSBI) did not change significantly during the course of this study. The discrepancy between the findings of the G.I. versus the mSBI have been observed in many studies [33]. The GI is based on two of the characteristic signs of inflammation—swelling (edema) and redness as well as bleeding. The mSBI is more strongly focused on bleeding and edema [33]. One study directly comparing data from the 2 indices in the same patient group determined that although gingival edema and change in color are often found together, bleeding can occur independently of edema [33]. These data underline the importance of more extensive and comprehensive studies that are carefully controlled over adequate periods of time to ensure maximum validity for the data obtained.

### 3.4. Saliva

Salivary volume, pH, and buffering capacity were recorded at the beginning and end of each study arm. Buffering measurements were made using GC Saliva-Check Buffer Kit.

#### 3.4.1. Saliva Volume

The volume of saliva collected over 5 minutes in subjects with xerostomia was approximately doubled at the end of the test and control study arms, representing a statistically significant increase in each case (*p* < 0.05) (Table 1). In the study arm with no intervention, saliva production did not change significantly (*p* > 0.1). The transient increase in saliva production is similar to that reported in other studies using similar products [7, 34].

#### 3.4.2. Salivary pH

pH values did not differ significantly between study arms and timepoints (*p* < 0.05) (Table 2).

#### 3.4.3. Salivary Buffering Capacity

Salivary pH buffering performance improved significantly in test and control arms of the study (*p* < 0.05). No change was observed in the arm when no intervention was used (*p* > 0.1) (Table 3).

Overall, this pilot study demonstrated that a novel dry mouth intervention has similar effects on plaque presence, salivary pH and buffering capacity, gingival health, and tooth sensitivity, as well as intraoral comfort and function as those of an existing OTC remedy [35–37]. The performance of the OTC remedy identified in this study resembled that documented in previous studies [35–37] and is significantly better than no intervention. Further studies are required in more patients and over a longer period of time.

## 4. Conclusions

In this first pilot study, the hypothesis was confirmed that a novel dry mouth intervention has similar effects as those of an existing OTC remedy and is significantly better than no intervention. Further studies are required in more patients and over a longer period of time.

## Figures and Tables

**Table 1:** Mean saliva volume (SD) collected over 5 minutes in subjects at the beginning and end of each study arm.

Baseline (ml (SD))	No intervention (ml (SD))	Test (ml (SD))	Control (ml (SD))
2.25 ml (0.35)	2.64 ml (0.27)	5.45 ml (0.48)	5.02 ml (0.42)

**Table 2:** Mean salivary pH at baseline and at the end of each study arm.

Baseline pH (SD)	No intervention pH (SD)	Test pH (SD)	Control pH (SD)
7.25 (0.75)	7.13 (0.67)	7.20 (0.68)	7.25 (0.62)

**Table 3:** pH buffering capacity at (i) baseline and (ii) at the end of each study arm.

	Baseline	None	Test	Control
pH buffering	Low (10/10 subjects)	Low (10/10 subjects)	Normal (8/10 subjects) low (2/10 subjects)	Normal (8/10 subjects) low (2/10 subjects)

## Data Availability

The data generated from this study include identifiable personal health information. Thus, they cannot be released for ethical and privacy reasons. Moreover, the datasets used to support this study are currently under embargo, while the research findings are commercialized. Requests for data, 12 months after initial publication, will be considered upon request with permission of UCI IRB.
